# 2D projection image reconstruction for field free line single-sided magnetic particle imaging scanner: simulation studies

**DOI:** 10.18416/ijmpi.2021.2104001

**Published:** 2021-04-27

**Authors:** Carlos Chinchilla, Chris McDonough, Amanuel Negash, Jason Pagan, Alexey Tonyushkin

**Affiliations:** a Physics Department, University of Massachusetts Boston, Boston, USA; b Medical Engineering Science, University of Lübeck, Lübeck, Germany

## Abstract

Magnetic Particle Imaging is an imaging modality that exploits the nonlinear response of superparamagnetic iron oxide nanoparticles to a time-varying magnetic field. In the past years, various scanner topologies have been proposed, which includes a single-sided scanner. Such a scanner features all its hardware located on one side, offering accessibility without limitations due to the size of the object of interest. In this paper, we present a proof of concept image reconstruction simulation studies for a single-sided field-free line scanner utilizing non-uniform magnetic fields. Specifically, we implemented a filtered backprojection algorithm allowing a 2D image reconstruction over a field of view of 4 × 4 cm^2^ with a spatial resolution of up to 2 mm for noiseless case.

## I. Introduction

Magnetic Particle Imaging (MPI) is a novel biomedical imaging modality, which promises a higher sensitivity among the existing medical imaging technologies [1, 2]. MPI features various promising aspects, such as high temporal resolution, no ionizing radiation, and no attenuation by tissues. Various groups have proved that MPI can be exploited in biomedical applications such as cell tracking [3–5], cancer imaging [6], and hyperthermia [7–9]. MPI detects the presence of superparamagnetic iron oxide nanoparticles (SPIONs), which are excited and spatially encoded with a distinct set of electromagnetic coils, depending on the scanner topology. To date, several scanner topologies have been proposed [1, 10–12]. Among the notable MPI developments is a single-sided MPI scanner, which was first introduced for field-free point (FFP) field topology [11]. The single-sided MPI scanner confines all the hardware to one side of the device, which is beneficial, as it provides the patient with unrestricted access to the scanning area [13], although, with a limited penetration depth [14]. Unlike the original single-sided MPI scanner, which utilizes an FFP [13, 15, 16], we designed a single-sided device with a field-free line (FFL) [17, 18] with a promise of a higher sensitivity [10] and more robust image reconstruction [19].

To date, we have built a prototype of permanent magnet selection coils for the FFL scanner [20] and all electromagnet prototype of a single-sided FFL device [21]. Using the latter device we have demonstrated a spatial trajectory encoding and performed SPIONs sensitivity studies [22]. However, imaging algorithms yet need to be devised and experimentally demonstrated for this device. In this paper we present the first proof of principal simulation studies of 2D image reconstruction for a single-sided FFL scanner with non-uniform fields. Previously, such fields were shown to cause imaging artifacts [23]. Here, we show simulation studies by means of filtered backprojection (FBP) image reconstruction technique [24], which is specifically tailored for the prototype of single-sided MPI scanner that uses an FFL for spatial encoding [21] and characterize its imaging performance.

## II. Theory

### II.I. Scanner Design and Signal Calculations

**Figure 1:**
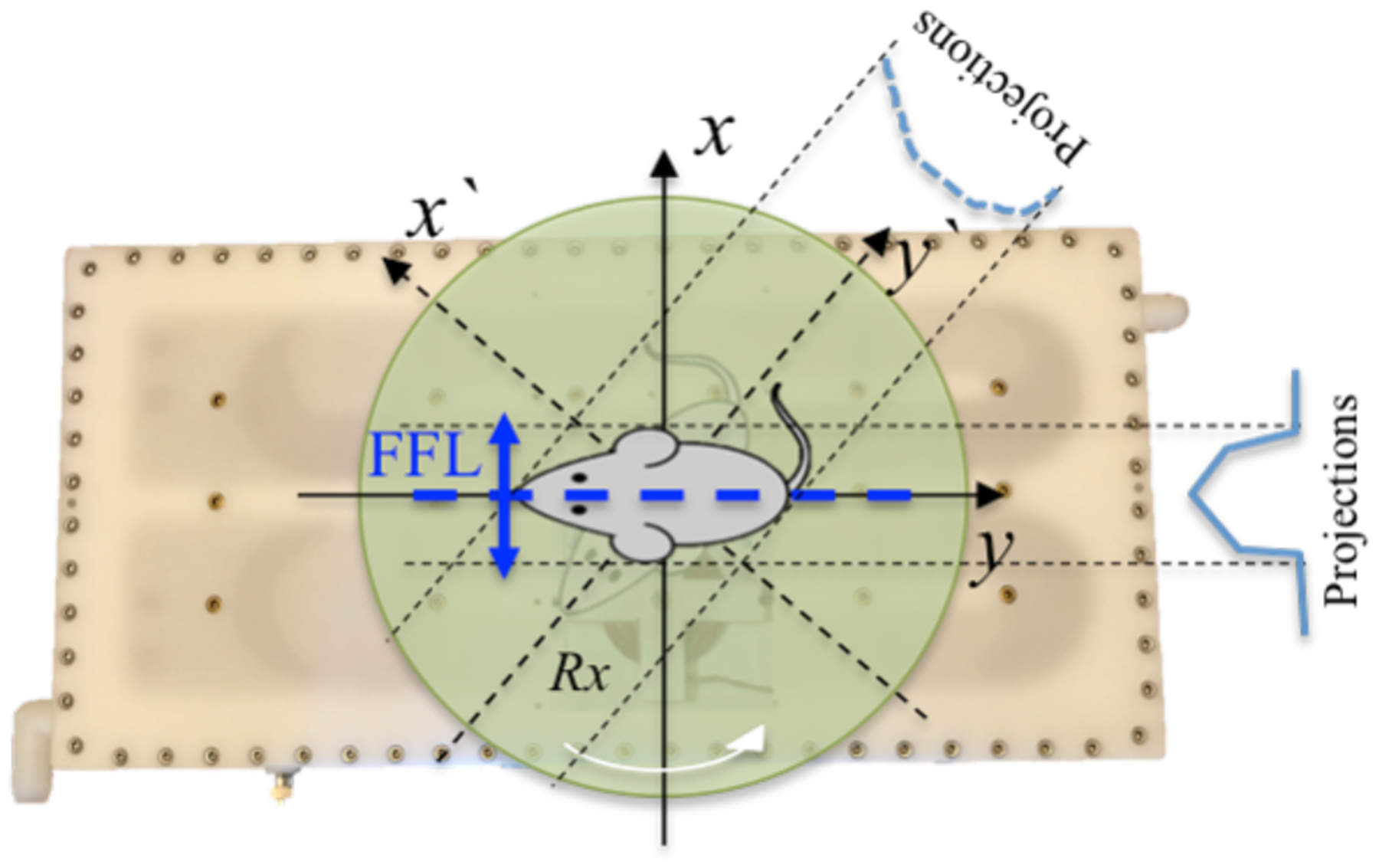
Single-sided MPI scanner. The FFL is generated through the selection coils along the y-axis and translated along x-axis while the subject turntable is mechanically rotated for 2D spatial encoding at a fixed height.

The single-sided scanner’s schematic diagram is shown in Figure 1. In our approach, the sample is placed on a rotating platform, which is located above the electromagnetic coils. In order to collect data at different angles, the platform is mechanically rotated to a discrete angle position. At each angle, the FFL is electronically translated along the *x*-axis in discrete steps incorporating the desired field of view (FOV) to form a single projection. Hence, a sinogram can be generated, which is used to reconstruct an image from the measured signals with the FBP method [25].

**Figure 2:**
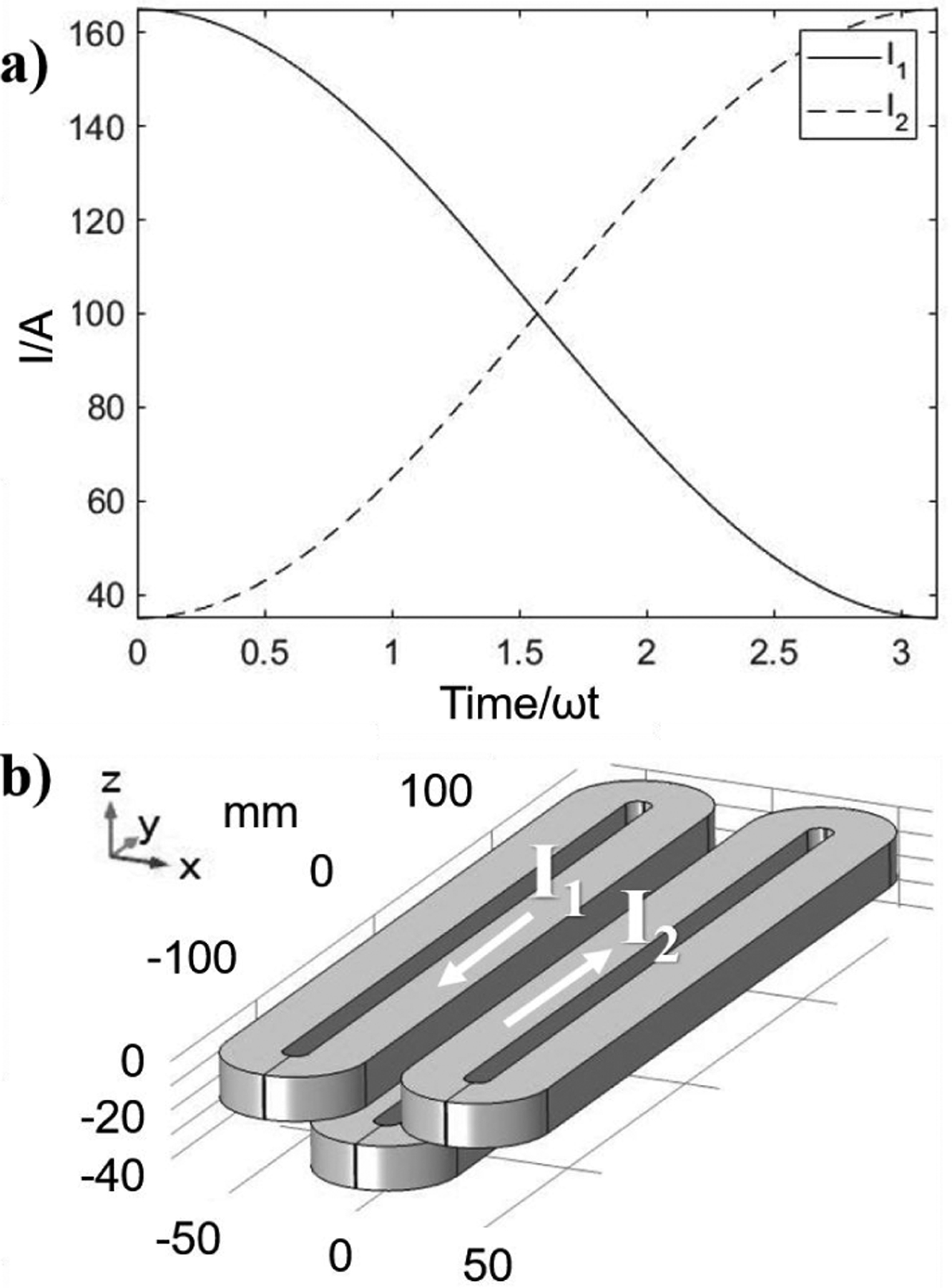
a) Current patterns applied to the selection coils. b) Coil system used in simulations. Upper and lower coils generate selection and drive fields, respectively.

The diagram of the coils used in our single-sided scanner and the current pattern to encode the FFL trajectory are shown in Figure 2. The two selection field (SF) and the drive field (DF) coils are located near the surface of the device in two layers as shown in Figure 2(b). The currents applied to the selection coils to generate the FFL along the *y*-direction and shift it along the *x*-direction are governed by the following equations (see Figure 2(a)):

(1) $$I_{1}=I_{0}\left(1+\alpha \text{ cos}\left(\omega t\right)\right),\;I_{2}=I_{0}\left(1-\alpha \text{ cos}\left(\omega t\right)\right),$$

where *I*_0_ is the current amplitude that defines the strength of the gradient of the magnetic field. The scaling factor *α* corresponds to the spatial range covered by the FFL defining the field-of-view (FOV), as it determines the gap between the maximum values of the currents, and *ωt* is the unitless time characterised by the angular frequency parameter *ω* [26]. Varying the currents in the two SF coils according to (1) translates the FFL in *xy*-plane, providing 2D spatial encoding.

The DF coil generates the excitation field at the frequency *f*_*D*_. In our implementation the non-uniform DF, most generally, is given by the following expression:

(2) $${\vec{B}}_{\text{Drive}}\left(x,z,t\right)=\left(B_{x}^{D}\left(x,z\right)\hat{x}+B_{z}^{D}\left(x,z\right)\hat{z}\right)\text{sin}\left(2\pi f_{D}t\right),$$

where $B_{x,z}^{D}$ determine the non-uniform amplitudes of the magnetic field. In addition to generating the AC excitation field, the drive coil is used to produce a variable bias offset field that is required for the dynamic FFL trajectory correction, as described in Section II.II. The offset field shares the same spatial component as in (2) but doesn’t have a fast sinusoidal time component.

The SF for the FFL generator magnet in the vicinity of the FFL is described as follows:

(3) $${\vec{B}}_{\text{Sel}}\left(x,y,z\right)=G\cdot \vec{r}=\left(\begin{array}{ccc} G_{xx} & 0 & 0\\ 0 & 0 & 0\\ 0 & 0 & G_{zz} \end{array}\right)\left(\begin{array}{l} x\\ y\\ z \end{array}\right),$$

where *G*_*xx*_ and *G*_*zz*_ define the strength of the gradient magnetic field along the *x* and *z* directions, respectively. For both *x* and *z* directions holds *G*_*xx*_ = −*G*_*zz*_. The total magnetic field used herein is the superposition of drive, selection, and an offset field:

(4) $${\vec{B}}_{\text{Total}}\left(x,y,z,t\right)={\vec{B}}_{\text{Drive}}\left(x,z,t\right)+{\vec{B}}_{\text{Sel}}\left(x,y,z\right)+{\vec{B}}_{\text{Offset}}\left(x,z\right).$$

The magnetization can be mathematically described in the frame of the Langevin model for the ideal SPIONs as

(5) $$\vec{M}\left(x,y,z,t\right)=c\left(x,y,z\right)mL\left(\beta \left\|{\vec{B}}_{\text{Total}}\right\|\right)\frac{{\vec{B}}_{\text{Total}}}{\left\|{\vec{B}}_{\text{Total}}\right\|},$$

where *c*(*x*, *y*, *z*) describes the spatial distribution of the magnetic nanoparticles and $\beta =\frac{m}{k_{B}T}$ is the constant that depends on the dipole moment *m* of the SPION, on *k*_*B*_ - the Boltzmann’s constant, and on *T* the temperature of the particle in units of Kelvin.

As the magnetization oscillates due to the drive field, a receive coil placed on the surface of the device is used to measure an induced voltage according to Faraday’s law. The signal voltage is defined as following:

(6) $$v\left(t\right)=\int \frac{\partial }{\partial t}\vec{M}\left(x,y,z,t\right)\cdot {\vec{B}}_{Rx}\left(x,y,z\right)\text{dxdydz}.$$

The first term in (6) defines the time derivative of the magnetization of the SPION; ${\vec{B}}_{Rx}$ is the sensitivity profile of the receive coil, which is defined as the magnetic field produced by a unit current. During translation of the FFL, a voltage signal will be produced for every discrete position of the FFL as the SPIONs are excited with multiple cycles of the drive field.

### II.II. FFL Trajectory Correction

For spatial encoding of a large FOV in *xy-*plane and selection of the imaging plane it is important to precisely control the position of the FFL in a linear fashion [25]. The quasi-static position of the FFL along *x*-direction can be shifted by adjusting the currents through the two SF coils according to (1), whereas the height of the FFL is altered by applying the bias offset field ${\vec{B}}_{\text{Offset}}\left(x,z\right)$ generated by the DF coil. By combining the three currents varied in time it is possible to provide a flat trajectory in *xy* imaging plane at the fixed height as demonstrated experimentally [21]. Here we provide the algorithm of the trajectory correction for linear spatial encoding.

We first model the trajectory provided by the current pattern in Figure 2. In order to fit the position of the FFL versus the currents *I*_1_, *I*_2_ we introduce shift and offset parameters as *S* and *O*, correspondingly, which are defined below:

(7) $$S=\frac{I_{1}-I_{2}}{I_{1}+I_{2}},$$

(8) $$O=\frac{I_{\text{drive}}}{I_{1}+I_{2}}.$$

**Figure 3:**
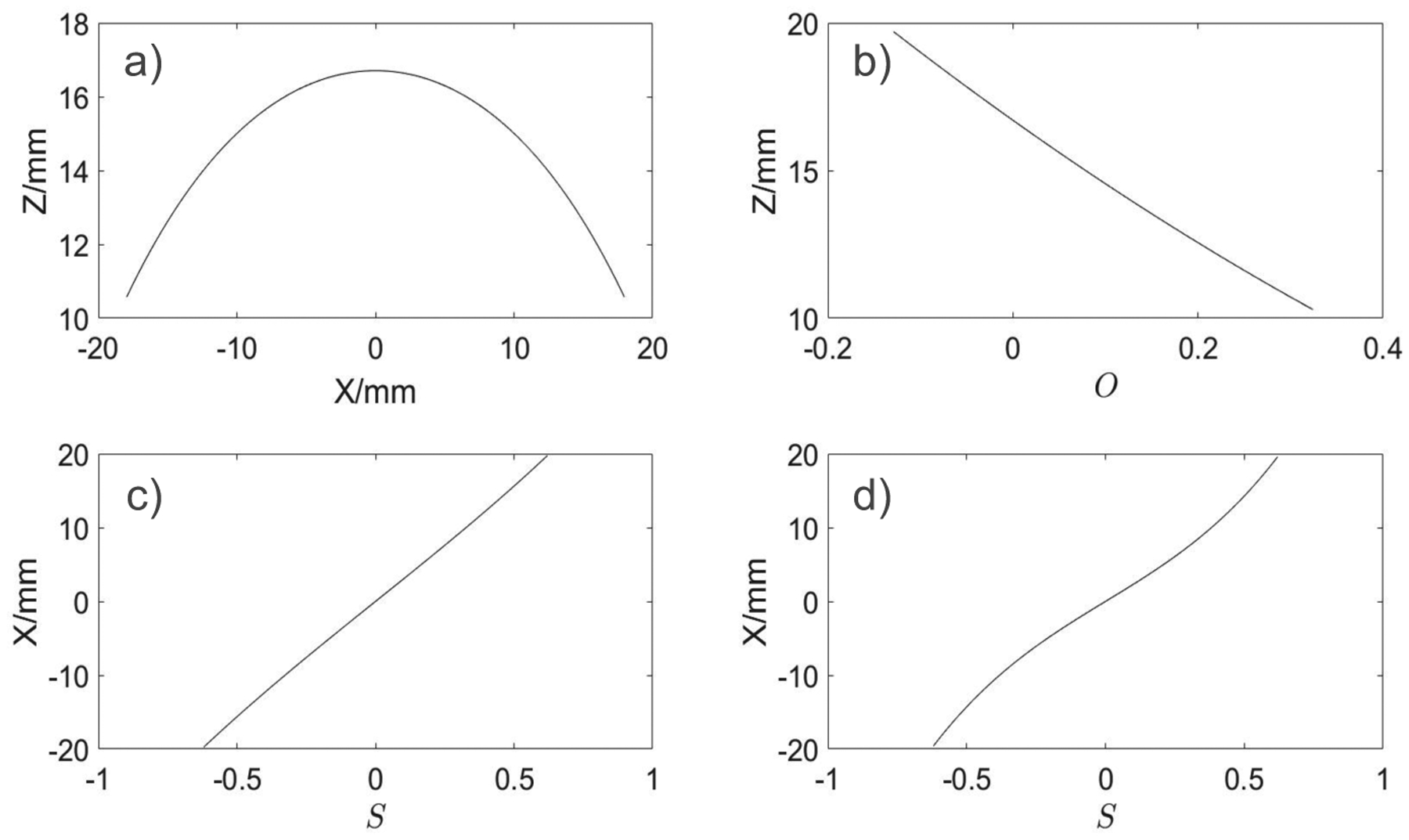
FFL-trajectory correction. a) Uncorrected non-flat *z*_*FFL*_ (*x*) trajectory; b) height correction due to offset current *O* in DF coil; c) *x*_*FFL*_ (*S*) dependence for *O* = 0; d) *x*_*FFL*_ (*S*) dependence for *O* ≠ 0.

First we consider FFL trajectory without the drive field. This was done by simulating our fields while varying *I*_1_ and *I*_2_, which is the same as varying the *S* parameter. Upon varying *S* we observe that the FFL travels in an arc, which can be fit by a 6^*th*^ order polynomial, this can be seen in Figure 3(a).

To control the FFL height we apply a bias field generated by the DF coil. We determine the relationship between the height of the FFL and the offset parameter at a shift of zero. This was done by simulating the fields with *I*_1_ = *I*_2_ and varying *I*_*drive*_. This fit can be seen in Figure 3(b) and is represented by the following equation:

(9) $$z=A_{2}O^{2}+A_{1}O+A_{0}.$$

The constants *A*_*i*_ for *i* = 0, 1, 2 vary at different *S*, where *A*_0_*S* corresponds to the arc when the FFL travels without drive current applied. The relationship between these constants and *S* were determined by fitting *z* versus *O* in the same way as we did above but at different *S*. Solving (9) provides *I*_*drive*_ corresponding to the FFL’s height of *z*:

(10) $$I_{\text{drive}}\left(z,I_{1},I_{2}\right)=\left(I_{1}+I_{2}\right)\times \left(-A_{1}S-\sqrt{A_{1}S^{2}-4A_{2}S\left(A_{0}S-z\right)}\right)/2A_{2}S.$$

Next, we control the *x* position of the FFL while at the same time adjusting its height. Simulations were run to determine the relationship between *x* and *S*. It can be seen in Figure 3(c) that in the presence of no drive field *x* has a linear relationship with *S*. However, this linear dependence breaks when we apply an offset field to include height correction. In the presence of the offset field the relationship between the *x*-position of the FFL and *S* can be represented by a 3^*rd*^ order polynomial as following (see also Figure 3(d)):

(11) $$x=B_{3}S^{3}+B_{2}S^{2}+B_{1}S+B_{0}.$$

The constants *B*_*i*_ for *i* = 0, 1, 2, 3 are functions of the height *z*. Applying this fit at different heights allows us to model these functions as polynomials. So the final equation becomes:

(12) $$B_{3}\left(z\right)S^{3}+B_{2}\left(z\right)S^{2}+B_{1}\left(z\right)S+B_{0}\left(z\right)-x=0.$$

Thus *S* can be determined as the real root of the (12).

## III. Material and Methods

### III.I. Simulation of the Coil System

The two racetrack SF coils simulated to generate the FFL have a length of 24.5 cm, a width of 54 mm, a height of 18.3 mm, 26 turns, a core gap of 10 mm and a gap between the selection coils of 11 mm. The conductor wire is made of copper with a rectangular cross-section of 1 × 1 mm^2^. The upper surface of the drive coil is located 4 mm from the lower surface of the selection coils. The DF coil is centered between the SF coils. For the receive coil we utilize a planar circular coil [22] located at the center on the surface of the scanner. Computation of the Biot-Savart law for all magnetic fields and image reconstruction were performed with MATLAB (Mathworks, Natick, MA).

**Figure 4:**
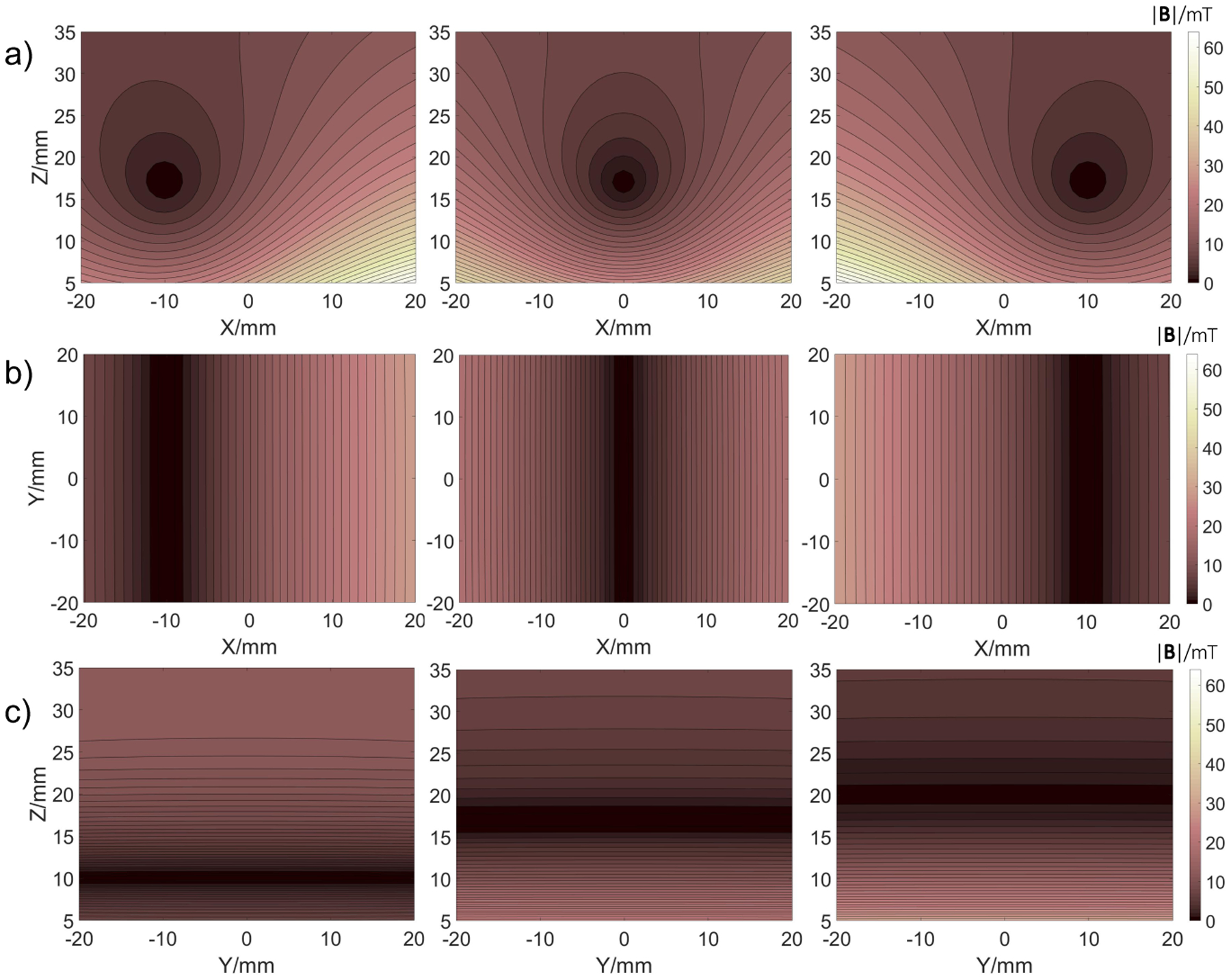
Simulated magnetic field contour plots |**B|/**mT. Rows: a) xz- and b) xy- planes showing FFL translations along x-axis with a linear corrected trajectory at the height z=17 mm; c) yz-plane showing FFL at x=0 and various slices z=10 mm, z=17 mm, z=20 mm.

The simulated selection magnetic fields are shown in Figure 4. For this simulation, a reference current *I*_0_ = 77 A was chosen corresponding to the gradient of 0.77 T/m at the static height of 17 mm above the surface of the scanner. Translation of the FFL, as shown in Figure 4(a, b) for *x* = ±10 mm, was simulated by applying currents *I*_1_ and *I*_2_ in the SF coils according to the pattern in Figure 2(a) and the offset current *O* in DF coil to correct the trajectory as described in Section II.II. In addition, three different reconstruction slices were considered as shown in Figure 4(c) for the FFL at x=0 and the heights: *z* = 10, 17, 20 mm. All the fields are uniformly rotated with a discrete angle with respect to the phantom (see Figure 1). Consequently, that allows data to be acquired and reconstructed to image the sample.

**Figure 5:**
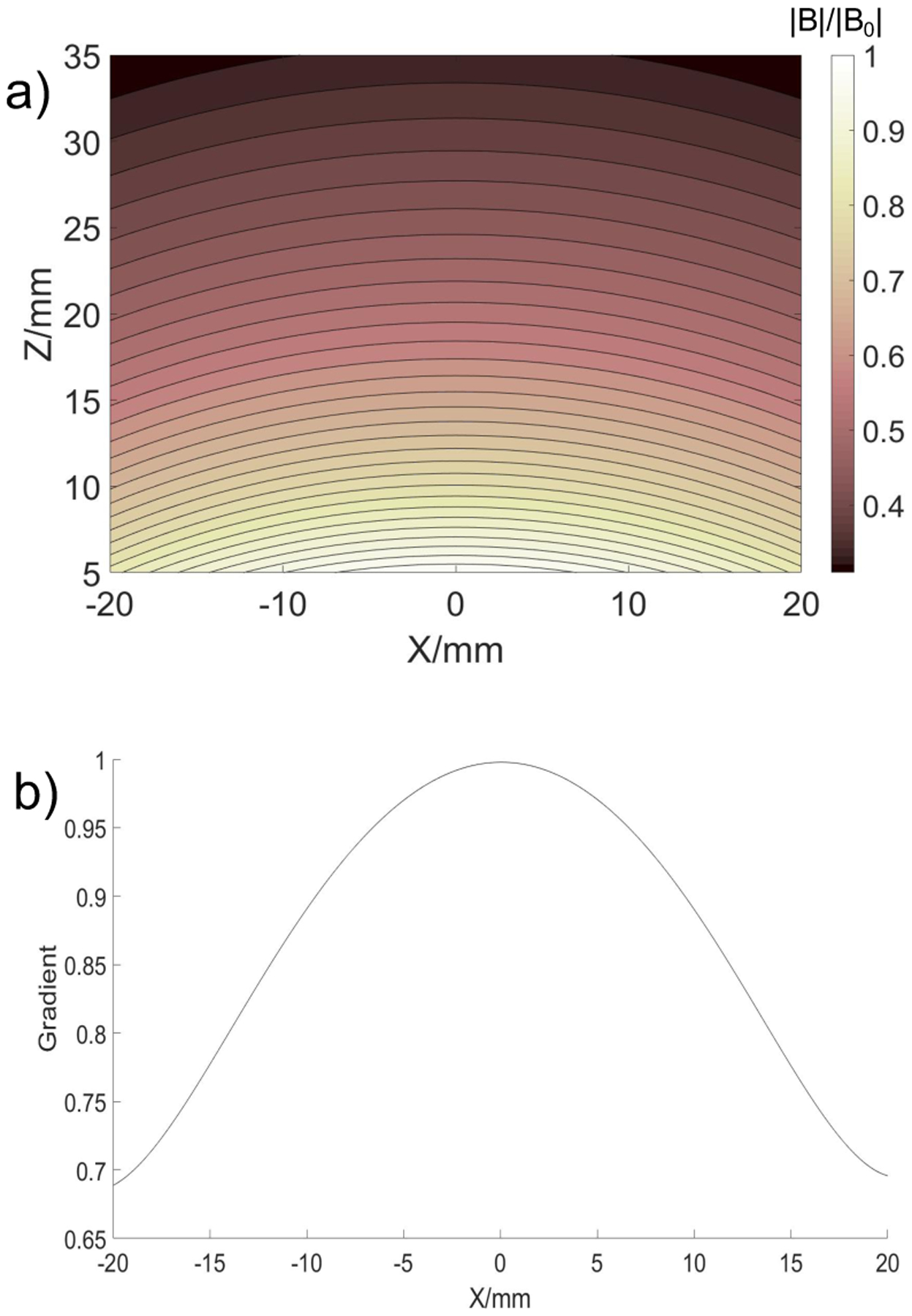
a) Simulated drive magnetic field contour plots |**B**|/|**B**_**0**_| in zx-plane; b) normalized gradient of the magnetic field versus the FFL scan position along x-axis.

The simulated normalized drive field is shown in Figure 5(a). The contour plot shows the non-uniform nature of the drive field in the full FOV = 4 cm^2^. The combined effect of SF and DF on the gradient of the magnetic field is shown in Figure 5(b), where we plotted the normalized gradient as the FFL is scanned along x-axis spanning the full FOV. Thus, the field gradient drops at the edges by 10% for 20 mm FOV and 30% for 40 mm FOV.

### III.II. Image Reconstruction

The single-sided apparatus allows electronic shifting and changing height of the FFL in *xy*-plane and *z*-axis respectively, while the rotation of the FFL is done mechanically. In these simulations of the image reconstruction we adapt the FBP method, where the data are collected in discrete steps for each translation of the FFL [27]. The FBP method is implemented as follows: for every value of the ratio of currents *S* (see (7)), a position of the FFL is calculated in the given FOV at the specified plane. For every position of the FFL, multiple cycles of the drive field excite the nanoparticles inducing a voltage signal from (6) and hence, obtaining a projection point. This process is repeated for the number of shifts specified for the simulation to form a projection at a given angle. Simulating projections from a discrete number of angles in the range of 0°-180° enables to measure a sinogram. Once a sinogram has been obtained, filtering in the Fourier space can be performed with the desired filter. In this work, we filter the projections obtained with the Hann, Cosine, and Shepp-Logan filters. These filters have been chosen, as they exhibit different behavior in the frequency space. Lastly, filtered projections are smeared back into the image matrix to render a 2D image. To mimic the current experimental scheme, in this work, we consider the third harmonic of the signal.

Several image reconstruction studies were performed in different planes at heights of 10, 17, and 20 mm to study imaging properties of the system. In all studies we simulated two concentration dots 1 × 1 mm^2^ phantoms with undiluted (5 mg/ml) SPION of core-diameter *d*_*c*_ = 25 nm with the magnetization behavior according to the adiabatic Langevin model [25]. In all the studies, a sinusoidal drive field with the frequency of 25 kHz was used with the drive field amplitude of 5 mT at the surface.

To characterize the spatial resolution featured by our system we utilize the contrast function ***C*** according to

(13) $$C=\frac{I_{Max}-I_{Min}}{I_{Max}+I_{Min}},$$

where *I*_*Max*_ and *I*_*Min*_ being the maximum and minimum of the intensity values of the image. Contrast greater than zero indicates capability of the system to spatially resolve objects.

In the first phantom study the two-dot were separated by 7 mm, the reconstruction was performed with FOV=4 × 4 cm^2^ for various numbers of translations of the FFL and projections (0°-180°) with Hann filter. Based on the results, we used 81 shifts and 54 rotations for all consecutive simulation studies.

In the second study we varied the distance separating the dot phantoms to determine the achievable spatial resolution of the system with ***C*** *>* 0. Moreover, the second study was carried out at three heights: 10, 17, and 20 mm with FOV=2 × 2 cm^2^. In each plane the separations between the dots were as following: 2, 3, 4, and 5 mm. In all of the simulations the gradients at x=0 vary between 2.08 T/m, 1.25 T/m, 1.01 T/m for 10, 17, and 20 mm respectively. Contrast calculations were performed using (13).

Subsequently, a noise study was performed to give the insight into the dependence between the noise level and signal-to-noise-ratio (SNR). White Gaussian noise of different normalized values of −60 dB, −40 dB, −20 dB, and −10 dB with respect to the maximum signal was added in this study. It was simulated at an FFL height of 10 mm for fixed separation of 7 mm between the dots. In addition, we investigated the effect from three standard filters: Hann, Cosine, and Shepp-Logan.

Lastly, a phantom with “UMB” letters was reconstructed with FOV=4 × 4 cm^2^ at the height of 10 mm to provide proof of the feasibility of a 2D reconstruction of a complex phantom with relatively large FOV by this type of single-sided scanner.

## IV. Results and Discussion

**Figure 6:**
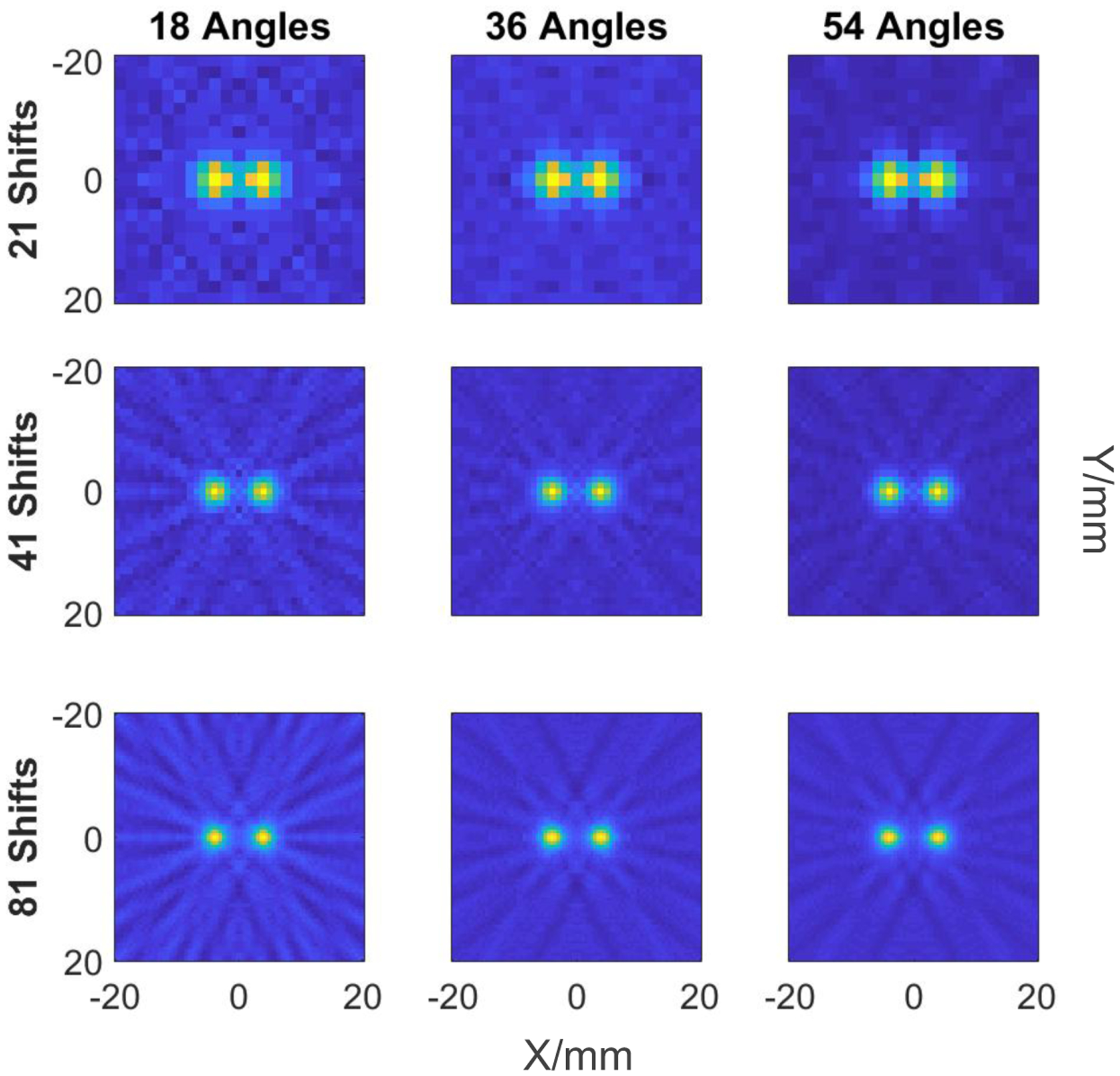
Reconstructed images of two dots 1 × 1 mm^2^ separated by 7 mm with Hann filter for 21, 41, and 81 FFL-translations (left to right columns); 18, 36, and 54 projections (top to bottom rows).

The results of the first study with various imaging parameters of FBP are shown in Figure 6, where columns represent number of rotations (18, 36, 54) and rows represent number of shifts (21, 41, 81). There is a clear improvement in the contrast with the increased number of shifts as seen for the maximum 81 shifts in the bottom row. The minimum estimated required number of rotations for the method is 18, however, increasing the number of rotations to 54 contributes to marginally better contrast with less streaking artifacts in the background. While higher number of rotations becomes impractical, these artifacts are phantom’s size dependent and go away for larger phantoms. The study shows that the conservative set of parameters, *e. g*., 36 angles and 41 shifts, may be sufficient in the experiment when the imaging time is important.

**Figure 7:**
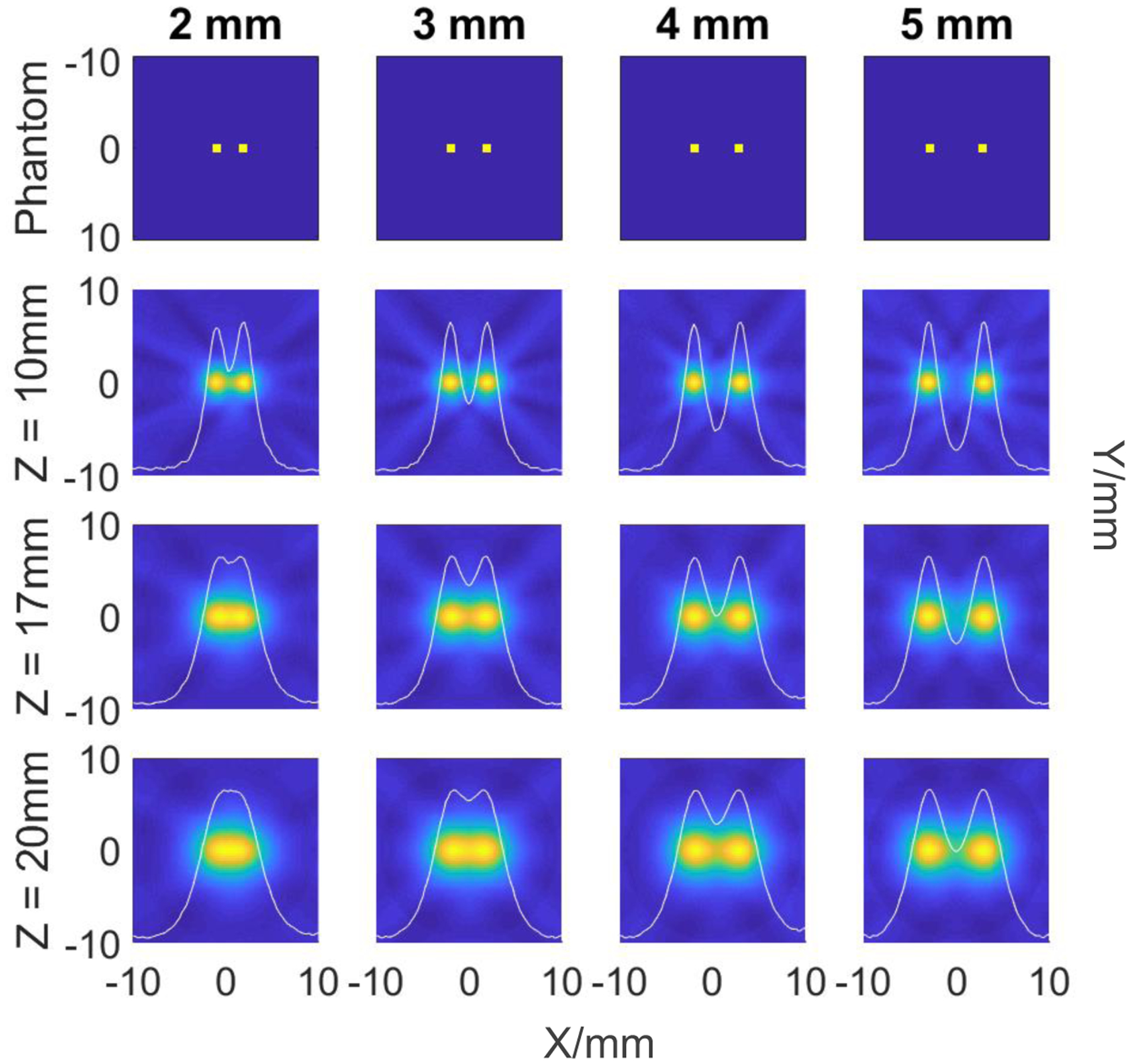
Reconstructed images using Hann filter with 81 FFL-translations and 54 projections. Each column represents a fixed distance between the two dots of SPIONs: 2, 3, 4, and 5 mm; and each row represents the height of the reconstructed plane: 10, 17, and 20 mm. First row represents the actual phantoms. All images are normalized to the same scale. Each image has an overlaid intensity cross-section along *x*-axis through the center of two dots.

Figure 7 shows the results of the simulations of the dot phantoms with various distances 2, 3, 4, and 5 mm between them at the three reconstructed planes: z=10, 17, and 20 mm. For each image we calculated the corresponding intensity cross-section along *x*-axis through the center of two dots. As seen in Figure 7, the simulated scanner can resolve up to 2 mm dot separation at a height of 10 mm according to the achieved contrast ***C*** = 0.16. However, the spatial resolution drops with the plane’s height so at the static height of *z* = 17 mm the resolution is 3 mm (***C*** = 0.11) and at *z* = 20 mm it becomes 4 mm (***C*** = 0.11). This is an inherent property of single-sided scanners where the SF gradient decreases with the height. In principle, the gradient drop can be eliminated provided the implementation of the additional coils as described in [17].

**Figure 8:**
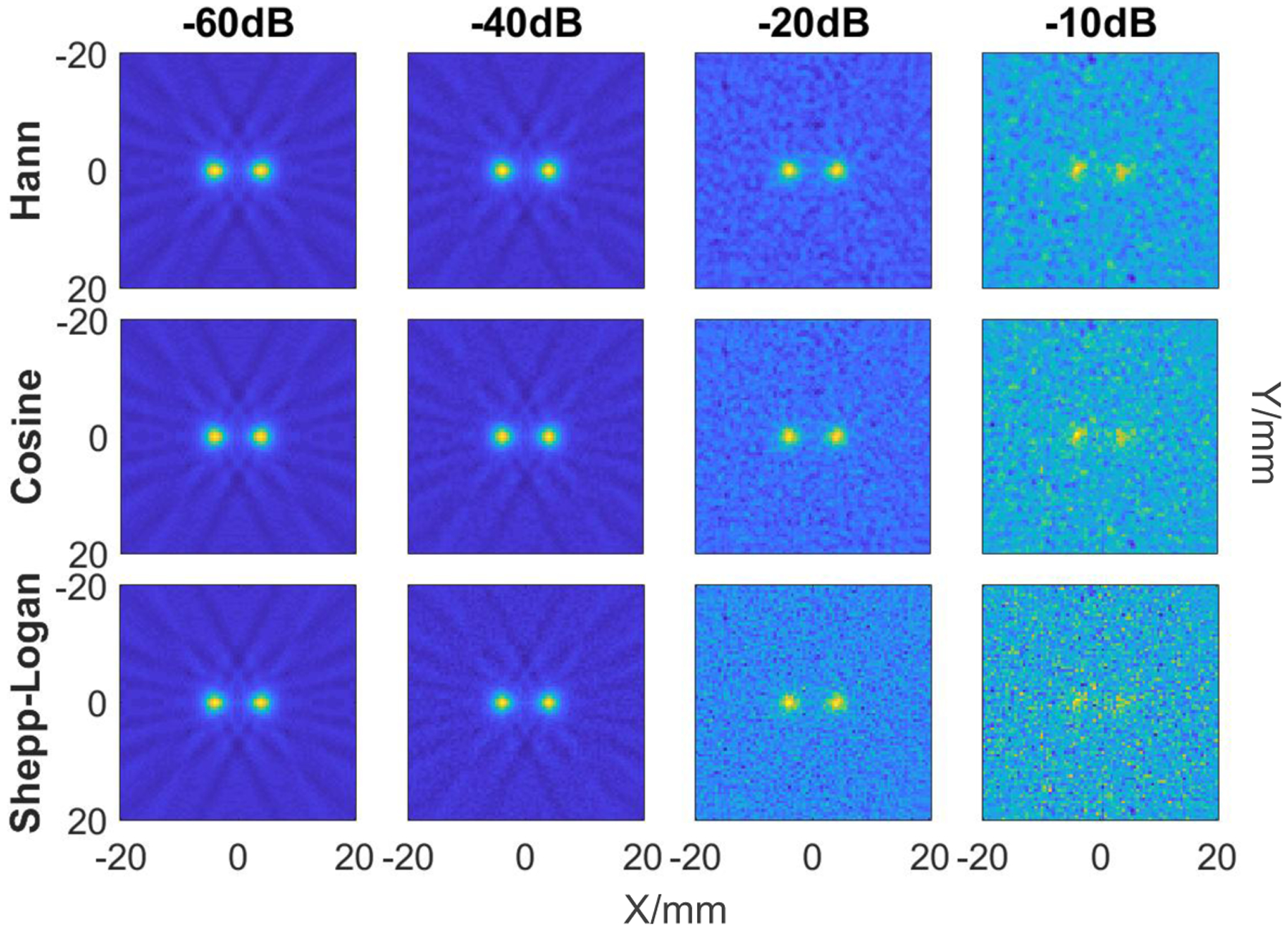
Reconstructed images of two dots 1 × 1 mm^2^ separated by 7 mm: Hann, Cosine and Shepp-Logan filters for 81 FFL-translations and 54 projections. Each column represents a different amount of noise level: −60dB, −40dB, −20dB, −10dB. SNR=73, 71, 16, 3 (first row left to right); 71, 64, 11, 2 (second row left to right); 69, 50, 6, 1 (third row left to right). All images are normalized to the same scale.

Figure 8 shows the results from the noise study. This study was performed with a magnetic field gradient of 2.08 T/m at a height of 10 mm and 7 mm separation between phantom dots. Different amount of noise level was included and three filters with sufficiently different behavior in Fourier space were considered. Results from Figure 8 demonstrate a decrease in SNR and contrast for increasing values of noise. Calculations show under these conditions, that the Hann filter delivers the best performance on SNR up to SNR=3 at the highest noise level, as seen in Figure 8. This effect is due to the behavior of the Hann filter in the frequency space, as high frequency values are not being strongly amplified, compared to the Cosine and Shepp-Logan filters, although Cosine filter provides the best contrast at moderate noise level.

**Figure 9:**
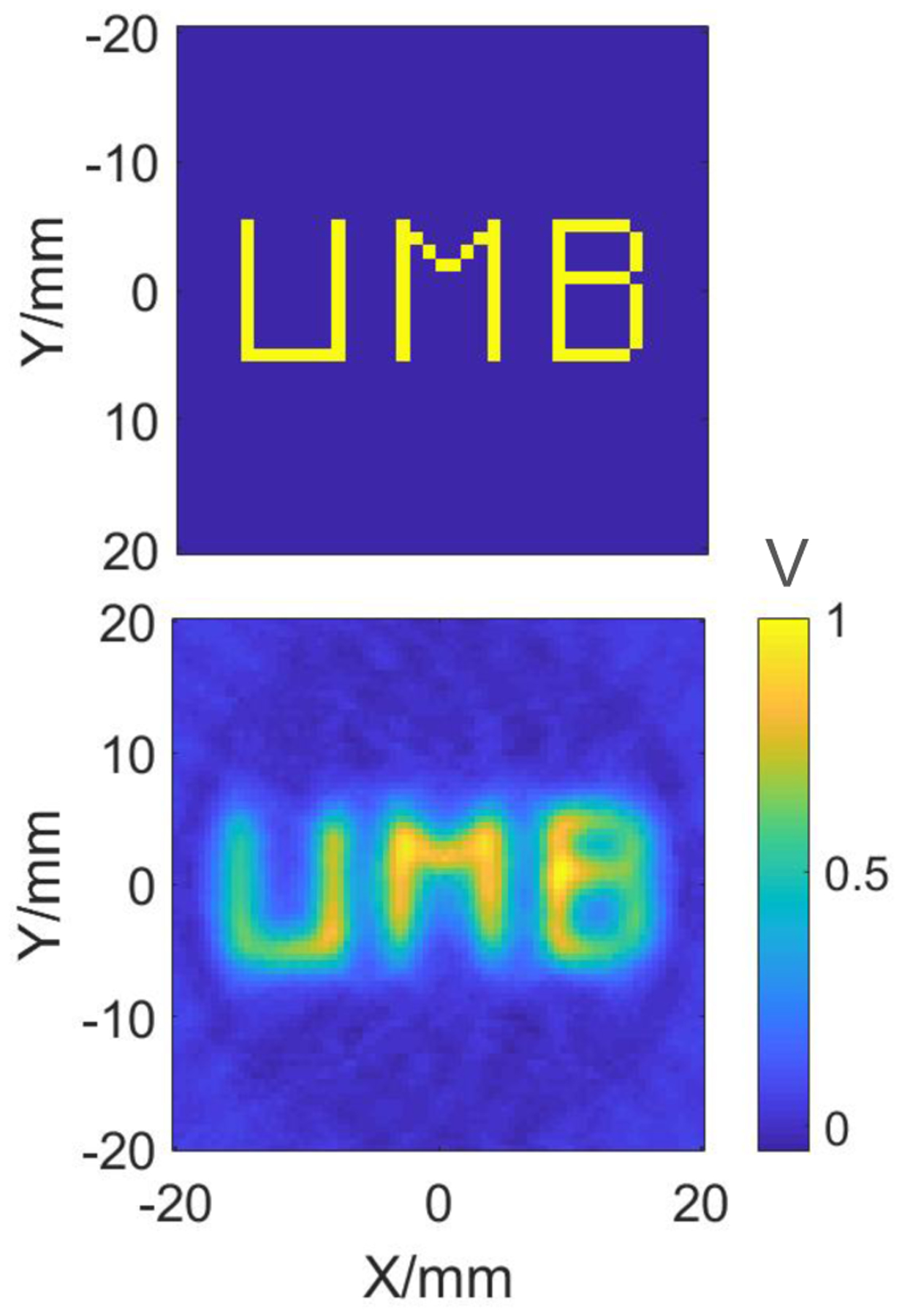
Image of a “UMB” phantom reconstructed over a FOV = 4 × 4 cm^2^ with Hann filter at *z* = 10 mm, 81 translations of the FFL and 54 projections. Here, the scale is represented by normalized voltage *V*.

Figure 9 shows the result for the simulated “UMB” phantom over a FOV of 4 × 4 cm^2^. This reconstruction was performed with Hann filter and 81 translations of the FFL and 54 projections at a fixed height of 10 mm above the surface of the scanner. Some drop off in the signal and there solution are observed at the edges of the FOV due to the non-linearity of the fields as shown in Figure 5. This is an inherent property of the current coil geometry due to the finite size of the drive coil. Higher uniformity over the large FOV could be achieved with the modified apparatus incorporating larger drive coil as well as implementing different receive coil schemes [22]. Nevertheless, the reconstructed image shows the feasibility of imaging of a complex phantom with an FBP reconstruction method over a relatively large FOV.

## V. Conclusions

We applied a projection-based FBP method to reconstruct images for a single-sided FFL MPI scanner with non-uniform field profiles. Several simulation studies were performed: variation of imaging parameters, spatial resolution, and noise studies. Subsequently, a FOV of 4 × 4 cm^2^ was simulated. Presented results show that 2D imaging with slice selection in the range of heights of *z* = 10−20 mm is feasible with the single-sided FFL device reaching the spatial resolution limit up to 2–4 mm for the noiseless case and up to FOV=4 × 4 cm^2^. In the future studies we will incorporate the SPION relaxation, extend the imaging into 3-D, and may consider advanced iterative reconstruction algorithms.
